# Supplementary material for: Conditional generative learning for medical image imputation
Source: Sci Rep. 2024 Jan 2;14:171. doi: 10.1038/s41598-023-50566-7 (PMC10762085; doi:10.1038/s41598-023-50566-7)
Supplement: Supplementary file 1 — Supplementary Information. [file 41598_2023_50566_MOESM1_ESM.pdf]

Table S1. Hyper-parameters for cWGAN.

Hyper-Parameter	Value/Type
Training Samples	12520
Latent space dimension (N_Z)	150
Batch size	15
Number of training epochs	800
Number of D updates per G update	4
ELU activation parameter	0.2
Regularization parameter	1e-7
Optimization technique	Adam ($\beta_1 = 0.5,\ \beta_2 = 0.9$)
Learning rate	0.001

Supplementary Notes

Supplementary Note 1. Linear Regression

The linear regression prediction of each image is found by solving an over-determined system using the ordinary least-squares method. The predicted image is then the linear combination of the three other images that are available. Therefore, for example, for the pre-contrast phase, the linear regression prediction R^1 is:

$$R^1 = \alpha * C + \beta * N + \gamma * E \tag{6}$$

where C, N and E are the true corticomedullary, nephrographic and excretory images respectively. The coefficients α, β, γ are determined by minimizing the mean squared error between the predicted image and real image across all samples in the training set (without data augementation). The final linear maps obtained are expressed as

$$R^1 = 0.259 * C + 0.266 * N + 0.242 * E, \tag{7}$$
$$R^2 = 0.300 * P + 0.502 * N + 0.172 * E, \tag{8}$$
$$R^3 = 0.309 * P + 0.504 * C + 0.294 * E, \tag{9}$$
$$R^4 = 0.380 * P + 0.234 * C + 0.398 * N. \tag{10}$$

Supplementary Note 2. Generator and Critic blocks

The generator and critic architectures are composed of several network blocks, as illustrated in Figure 6. The important components are explained below and while the training hyperparameters are listed in Table S1. In the generator we set $C = 32$, $k = 16$, $n = 3$, while in the critic we set $C = 20$, $k = 16$ and $n = 2$. The typical U-net uses Residual Blocks along with other neural networks. However, we have used Dense Blocks due to their superior performance. When it comes to the critic, a simple critic with downsampling has been used along with Dense Blocks. The standard layer normalization was used, unlike the generator where CIN was used. The exponential linear unit (ELU) was used in both the generator and the critic instead of the typical Leaky ReLU.

Convolution

The notation Conv(n, s, k) represents a 2D convolution operation in which k filters of size n are applied to the input tensor with a stride s. If n is greater than 1, reflective padding of width 1 is added to the input tensor in the spatial dimensions before the convolution. In case the third argument is not given, and the number of filters is set equal to the number of channels in the input tensor.

Conditional Instance Normalization (CIN)

The idea behind CIN[36] is that the latent variable z is injected into the generator at all levels of the generator (U-net). The CIN block takes as input the latent vector z and an intermediate tensor w of shape $H' \times W' \times C'$ and normalizes it along each channel $j = 1, \ldots, C'$ in the following way:

$$CIN(w,z)_j = \alpha(z)_j \bigotimes \left(\frac{w_j - \mu(w_j)}{\sigma(w_j)}\right) \bigoplus \beta(z_j) \tag{11}$$

where α: $R^{N_z} \to R^{C'}$ and β: $R^{N_z} \to R^{C'}$ are learnable convolution layers that take z as input. $\bigotimes$ and $\bigoplus$ are used to represent mathematical operations. $\bigotimes$ represents element-wise multiplication, which means that each element of one tensor is multiplied by the corresponding element of the other tensor. $\bigoplus$ represents summation in the channel direction, which means that the values in each channel of the tensors are summed together. $\mu(w_j)$ and $\sigma(w_j)$ compute the mean and fluctuations along the spatial directions, respectively. Therefore, each feature of the intermediate tensor is broken down into these mean and fluctuating components which are stochastic depending on the value of z through the learned layers α and β.

Dense Block

DenseBlocks are a type of building block used in convolutional neural networks for image classification tasks. They were introduced in the DenseNet architecture, which is a deep neural network that connects each layer to every other layer in a feed-forward fashion. We use the notation DenseBlock(k,n), where k represents the number of filters of size 3 and stride 1 in the 2D convolution, and n represents the number of sub-blocks. An example of a DenseBlock with n equal to 3 is shown in Figure S1. When the DenseBlock appears in the generator, it receives two inputs: an intermediate tensor w and a latent variable z. The latent variable is incorporated into the block using conditional instance normalization (CIN). However, when the DenseBlock appears in the critic, it only receives the intermediate tensor w as input. In this case, CIN is replaced with layer normalization.

In a DenseBlock, each layer receives the feature maps of all preceding layers as input, which are concatenated channel-wise. This means that the output of each layer in the DenseBlock is fed as input to all subsequent layers, allowing for highly efficient information flow throughout the network. By densely connecting the layers, DenseBlocks aim to reduce the vanishing gradient problem and improve gradient flow, which can lead to faster convergence during training.

Overall, DenseBlocks are a powerful tool for building highly efficient and accurate convolutional neural networks, especially for image classification tasks with limited data. Dense Blocks therefore work on preserving the feed-forward nature of the network as each layer obtains additional inputs from all the layers preceding it and then in itself passes feature-maps to all subsequent layers.

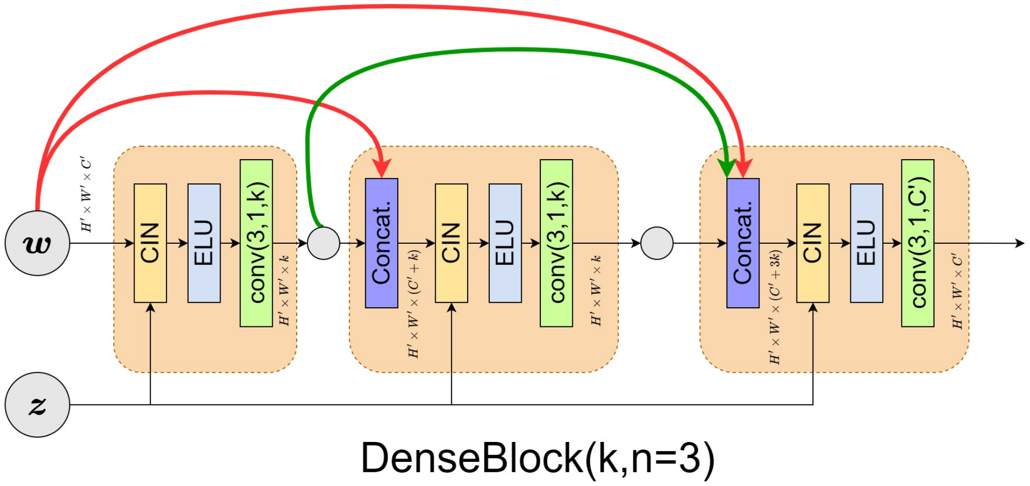

Figure S1. Architecture of a DenseBlock.

Down-Sampling Block

The down-sampling block (Figure S2) is designed to decrease the spatial resolution while simultaneously increasing the number of channels. This block comprises a convolution layer that produces an output with twice the number of channels as the input (q equal to 2), a 2D average pooling layer that decreases the spatial dimensions by a factor p of two, and a Dense block. The latent variable z is also an input when this block appears in the cGAN generator.

Up-Sampling Block

The up-sampling block (Figure S3) takes in the output $\mathbf{w}$ of the previous block along with the output $\tilde{\mathbf{w}}$ of a down-sampling block of the same spatial size via a skip connection. These tensors are then concatenated together in the channel dimension. Next, the up-sampling block carries out a convolution operation that divides the channel size by q equal to 2, followed by a 2D up-sampling operation that doubles the spatial dimension. Here, a 2D nearest neighbour interpolation increases the spatial resolution by a factor p of 2. Finally, the signal is passed through a Dense block.

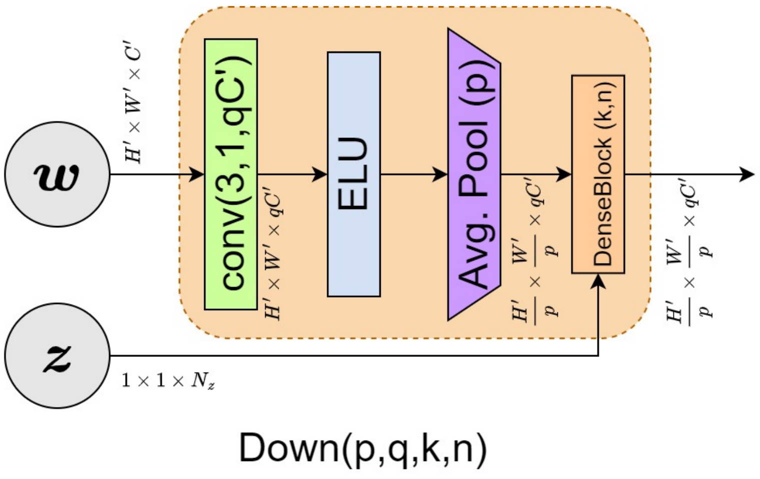

Figure S2. Architecture of a Down block.

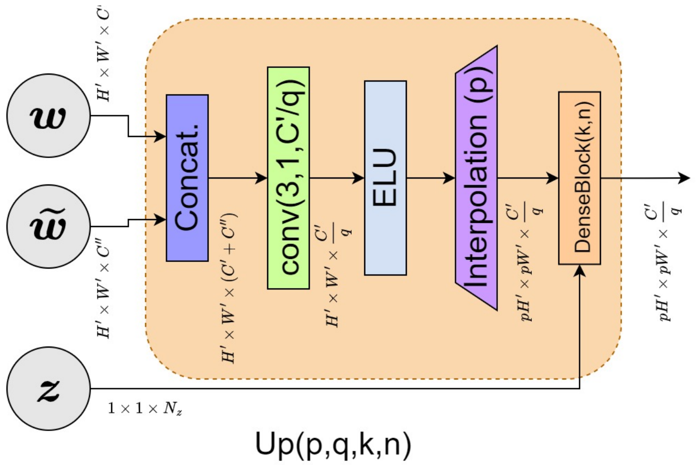

Figure S3. Architecture of an Up block.

Supplementary Note 3. The Gradient Penalty Term

The gradient penalty term is added to constrain the critic to be 1-Lipschitz on Ω_X.

$$D^*(G) = arg\max_D [L(G,D) - \lambda \mathop{\mathbb{E}}_{\varepsilon \sim U(0,1)} [(||\partial_1 D(h(x,y,z,\varepsilon),y)||_2 - 1)^2]] \tag{12}$$

The derivative in the gradient penalty term denotes the derivative with respect to the first argument of the critic. Also, $U(0,1)$ represents the uniform distribution on $[0,1]$. The function $h(x,y,z,\varepsilon)$ is:

$$h(x,y,z,\varepsilon) = \varepsilon x + (1-\varepsilon)G(z,y) \tag{13}$$

The gradient penalty parameter λ is set equal to 10.
